# Overview of the Motivation of Advance Care Planning: A Study from a Medical Center in Taiwan

**DOI:** 10.3390/ijerph18020417

**Published:** 2021-01-07

**Authors:** Yi-Jhen He, Ming-Hwai Lin, Jo-Lan Hsu, Bo-Ren Cheng, Tzeng-Ji Chen, Shinn-Jang Hwang

**Affiliations:** 1 Department of Family Medicine, Taipei Veterans General Hospital, Taipei 112, Taiwan; maybe8523@gmail.com (Y.-J.H.); mhlin@vghtpe.gov.tw (M.-H.L.); jlhsu2@gmail.com (J.-L.H.); tjchen@vghtpe.gov.tw (T.-J.C.); sjhwang@vghtpe.gov.tw (S.-J.H.); 2 School of Medicine, National Yang-Ming University, Taipei 112, Taiwan

**Keywords:** advance care planning, advance directives, health care agent, motivation, Taiwan

## Abstract

(1) Background: Since Taiwan’s Patient Right to Autonomy Act took effect in 2019, up to ten thousand declarants have participated in advance care planning (ACP) and have signed advance directives (ADs). Relative to the entire population of Taiwan, only a small percentage have completed ACP. This study sought to understand the motivations of Taiwanese who have participated in ACP, so as to increase the percentage of individuals participating in ACP and signing ADs; (2) Objectives: To understand the motivations that drive Taiwanese individuals to participate in ACP discussions.; (3) Methods: A retrospective secondary data analysis was performed in this study. The participants consisted of declarants who completed their ACP at a medical center in Taiwan in 2019; (4) Results: During the study period, 946 individuals completed their ACP. Of those declarants, 66.7% were over 60 years of age; 66.5% completed the process in groups of three or more; 49.5% completed their ACP free of charge; and 35 declarants had designated a health care agent (HCA). The declarants’ four main motivations for participating in ACP were “looking forward to dying with dignity,” “making end-of-life preparations,” “fear of being a social and economic burden on family members,” and “reluctance to let family members take on the responsibility of making decisions.” Furthermore, statistically significant differences were observed between the declarants in terms of gender, age, designation of an HCA, and motivations for participating in ACP. Females, declarants aged below 60 years, and declarants with a designated HCA tended to participate in ACP due to “reluctance to let family members to take on the responsibility of making decisions”. Males, declarants aged above 60 years, and declarants without an HCA came for ACP because of “fear of being a social and economic burden on family members”. (5) Conclusions: The main motivations of Taiwanese individuals who sought ACP were to die with dignity and to have an early understanding of end-of-life treatment and care models. Secondly, these individuals hoped that their families would not have to take on the responsibility of making decisions. They also did not want to impact their families socially and economically. In this regard, providing economic subsidies might enhance the Taiwanese public’s intentions to seek ACP discussions on their own initiative.

## 1. Introduction

Advance care planning (ACP) can assist patients, their families, and medical teams in communicating with one another when a patient’s disease has become terminal [1,2]. Following a comprehensive ACP session, a declarant can sign an advance directive (AD), which is a formal document in which they can express their expected medical care measures in advance before their physical and mental capacities become incapacitated [3,4,5]. Previous studies have shown that ACP can improve the quality of end-of-life care and reduce the possibility of institutionalization [6,7,8]. The process of implementing a good ACP session not only requires the declarant’s basic data (gender, age, etc.), but also their motivations for participating, background, and life expectations [9].

Death is a touchy subject [10,11]. In Taiwan, many patients do not attempt to express their intentions while they are still capable of making decisions for themselves. Consequently, when that capability becomes limited, whether as expected or unexpectedly, the medical interventions applied are instead determined by their families through discussions with their physicians [12]. This entails a failure to determine whether the patient would agree to the measures applied to them, which sometimes leads to physician–patient conflicts, confrontations, and legal disputes [13]. Hence, there is growing demand for discussions of end-of-life care models in advance. In 2016, Taiwan promulgated the Patient Right to Autonomy Act, the first of its kind in Asia [13,14]. The act aimed to ensure the patient’s right to be informed of his/her diagnosis and treatment options, and to make his/her own medical decisions, because critical diseases were often concealed to the patient in Taiwanese culture [14,15]. The act formally defined the objectives, legal significance, conditions, signing procedures, enforcement rules of ACP and ADs, and the role of the health care agent (HCA) [14,16]. In the ACP sessions, specific terminal conditions, life-sustaining treatments, artificial nutrition and hydration would be introduced to participants [14]. The ADs documented the participants’ thoughts about life-sustaining treatments, artificial nutrition and hydration if they faced those terminal conditions [14]. An HCA is a person who helps a declarant to express his or her wishes when his or her consciousness is lost or not clear enough to express the wishes [14,16]. A previous study revealed that up to 95% of critically ill patients lose their medical decision-making capacity toward their end of life [17]. The Act formally took effect in 2019. In addition to ensuring the patients’ palliative care rights, the Act also guarantees that medical professionals are able to administer medical treatments in compliance with patients’ wishes. As of September 2020, 16,360 declarants throughout Taiwan have completed the signing of their ADs [18].

Even with the passage of the Act, it remained difficult to initiate a discussion on ACP. From the perspectives of some physicians, barriers to implementing ACP in advance included not wanting a patient to give up hope, uncertainty of the right moment to initiate ACP, and the perception that patients should be the ones who initiate ACP, etc. [19,20]. The difficulty in initiating an ACP discussion also exists in other Asian cultures, such as Japan, South Korea, Hong Kong, and Singapore [21,22,23,24]. Confucianism greatly influences these cultures, and patient autonomy is less dominant when patients face end-of-life decision making [15].

This study aimed to explore the motivations for participating in ACP from the perspectives of declarants who have completed their ACPs. This study analyzed the one-year data of a major medical center in Taiwan in order to illuminate the motivations of Taiwanese people to participate in ACP and to analyze the factors that affect the declarants’ decision to participate in ACP and sign ADs on their own initiative. It is expected that this study could further promote ACP and AD signing among the Taiwanese population, thus allowing them to consider in advance their medical wishes when they have a terminal disease. Our study could assist in the promotion of AD signing in countries or cultures where death is a sensitive topic.

## 2. Materials and Methods

### 2.1. Data Collection

Data were collected at Taipei Veterans General Hospital (VGHTPE), a major medical center in Taiwan. The participants were declarants who completed their ACP in the outpatient department and wards from 6 January 2019 to 31 December 2019. As regulated in the Patient Right to Autonomy Act, only individuals who are 20 years of age and over and with full disposing capacity may sign an AD. Hence, all participants were over 20 years of age. During the process, counselors would record a declarant’s date of ACP, the time of ACP, gender, age, identity (veteran, low-income group, VGHTPE employee, or other), marital status, how many persons participate in each ACP session, accompanying relatives, date of uploading AD, whether the patient had designated an HCA, motivations for participating in ACP, interactions throughout the ACP process, and whether an AD was completed on the spot. The motivations were measured in a questionnaire with nine options, including “suffering from a disease”, “having a family member who is suffering from a disease”, “being single”, “reluctant to let family members to take on the responsibility of making decisions”, “looking forward to die with dignity”, “making end-of-life pre-arrangements”, “fear of being a social and economic burden to family members”, “media reports and dissemination”, and “other”. The category “other” was an open blank for participants to fill their own motivations different from other options. During data processing, all of this information was anonymized and delinked. The study was approved by the institutional review board (2019-11-005AC) of Taipei Veterans General Hospital, Taipei, Taiwan.

### 2.2. Advanced Care Planning in Taipei Veterans General Hospital

VGHTPE set up a dedicated “Advanced Care Planning Clinic”. Anyone, including patients and their families, can obtain information regarding the ACP from the leaflet, the electronic billboard in the hospital, the website and the Facebook account of the Advanced Care Planning Clinic. People who are willing to participate in ACP (declarants) can make appointment via the desk, the telephone or the website of the Advanced Care Planning Clinic. After the appointment is made, a member of the clinic will contact the declarants to provide prior reading materials (including articles and videos). Declarants first read the provided materials beforehand, participated in the ACP, and then signed and uploaded their ADs. At the beginning of the ACP session, all the participants completed the questionnaire about their ACP motivations. The participants could tick multiple options as they wanted. The counselors who participated in an ACP included a physician and a nurse, plus a psychologist or social worker. The time taken was measured in sessions, with each session predetermined to last for 90 min, which excludes preparations. In terms of the number of declarants and the cost of ACP, declarants who identified as a veteran, low-income group, or VGHTPE employees who participated in groups of three and above were fully reimbursed by the hospital; for a single participant, the cost is NT$3000; for two participants, the cost is NT$1500 per person; for three participants and above, the cost is NT$1000 per person (NT$1000 is about USD 34, based on the exchange rate on 25 September 2020). A maximum of six participants may participate in the same session [25].

### 2.3. Data Processing

This was a retrospective study. The following information was collected—the time of ACP; gender; age; marital status; type of participation; date of uploading AD; date of completing AD; and whether the declarant had designated an HCA. Afterwards, we analyzed the motivations for participating in ACP, whether the declarant had designated an HCA, the required cost, and the time required for the ACP process.

### 2.4. Statistics

Statistical analysis was performed using Office Excel (Microsoft, Redmond, WA, USA) and SPSS 22.0 (SPSS Inc., Chicago, IL, USA). The statistical methods included descriptive statistics, multiple-response sets, chi-squared tests, and Fisher’s exact test. Differences with a *p*-value smaller than 0.05 were defined as statistically significant.

## 3. Results

Throughout the research period, 946 declarants completed their ACP processes, 406 of which were male and 540 were female. The mean age of the declarants was 65.7 years, the median age was 67 years, and 66.7% of declarants were over 60 years of age. A majority (65.5%) of the declarants participated in ACP in groups of three or more, while only 10.6% of declarants came on their own. Almost half (49.5%) of the declarants completed their ACP free of charge. Overall, 63.0% of declarants completed all legal procedures (uploading their ADs) on the spot. A majority (90.2%) of the declarants uploaded their ADs within 90 days, while only a handful (3.9%) of them took over 90 days to do so. During data collection, 55 declarants did not formally sign their ADs after completing their ACP process. Among them, two declarants did not sign their ADs because cognitive impairment was found and neurologist evaluation was suggested during the process. Among the 946 declarants, only 35 (3.7%) of them had designated an HCA (Table 1).

In terms of the declarants’ motivations to participate in ACP, the top three motivations were “looking forward to dying with dignity,” “making end-of-life preparations,” and “fear of being a social and economic burden on family members.” For male declarants, the top three motivations were “looking forward to dying with dignity,” “making end-of-life preparations,” and “fear of being a social and economic burden on family members.” For female declarants, the top three motivations were “looking forward to dying with dignity,” “making end-of-life preparations,” and “reluctance to let family members to take on the responsibility of making decisions.” The declarants’ motivations were statistically different with regard to gender. In terms of age, overall, the top two motivations for declarants were “looking forward to dying with dignity” and “making end-of-life preparations.” The third most popular motivation for declarants aged below 60 years was “reluctance to let family members to take on the responsibility of making decisions,” while that for those above 60 years was “fear of being a social and economic burden on family members.” The declarants’ motivations were statistically different with regard to age. In terms of marital status, the top two motivations in general were also “looking forward to dying with dignity” and “making end-of-life preparations.” However, a higher percentage of those who were single participated in ACP on the basis of “being single.” The motivations of declarants were also statistically different in relation to marital status. There were 35 declarants (3.7%, *n* = 946) who had a designated HCA, and their top three motivations were “looking forward to dying with dignity,” “making end-of-life preparations,” and “reluctance to let family members to take on the responsibility of making decisions.” Their motivations had statistically significant differences with those of their counterparts without a designated HCA (Table 2).

Among the 35 declarants who had a designated HCA, 24 were female and 11 were male. The percentage of declarants with a designated HCA was 6.0% and 2.5%, respectively, among all declarants below and above 60 years of age. The percentage was 7.4% and 2.5%, respectively, among all single and married declarants. Compared to the declarants without a designated HCA, the differences were statistically significant in terms of age and marital status (Table 3).

The mean and median length of the ACP processes was 64 and 60 min, respectively. A total of 54 declarants had spent more time than the predetermined 90 min, with the mean and median length being 102 and 100 min, respectively. Among these 54 declarants, there were more females than males, and in relation to gender, statistically significant differences between the lengths of the ACP processes (exceeding or under 90 min) were observed. There were more declarants over the age of 60 who required more than 90 min of ACP compared to those under 60 years, but the percentage of those under 60 years who required more than 90 min of ACP was higher (7.9%), with a statistically significant difference. Furthermore, the percentage of declarants with a designated HCA who required over 90 min of ACP was higher, and the differences were statistically significant. In terms of cost, of the 54 declarants who took more than 90 min, four of them paid NT$1500 and 40 of them paid NT$1000 for their ACP (Table 4).

In terms of the cost of ACP, over half of the male declarants (63.8%) participated for free, while most of the female declarants paid for their ACPs (61.3%). Only 40% of declarants aged above 60 years paid for their ACPs, but over 70% of their counterparts below 60 years did so. The percentage of single declarants who paid for their ACPs was higher than that of married declarants. Therefore, with regard to paying for their ACPs, there were statistically significant differences among the declarants in terms of their gender, age, and marital status (Table 5).

## 4. Discussion

Our study yielded the following main findings: most of the declarants were female, aged over 60 years, and participated in their ACP session in groups of three or more; more than half of the declarants completed all of the procedures on the day of their ACP session; 90% of them completed the uploading of their ADs within 90 days; and only a small percentage (3.7%) of them had designated an HCA. Next, regardless of subgroups across all the declarants, the two primary motivations for participating in ACP were “looking forward to dying with dignity” and “making end-of-life preparations.” Thirdly, age had an influence on the declarants’ designation of an HCA. Fourth, a small proportion of the declarants had spent more time on their ACP process, most of whom were female, under 60 years of age, and had a designated HCA. Lastly, economic subsidies may increase the willingness of the public to seek ACP discussions.

According to a 2019 study, there were more female American patients who had completed their ACP process (54.4%), and the mean age of the patients was 81.4 years [26]. Our study indicated that the mean age of the declarants who had signed their ADs was 65.7 years. The large difference between the aforementioned study and our study could be attributed to the different patient groups. The participants in the aforementioned study were seriously ill patients above 65 years of age whose mean serious illness diagnosis was 1.4, and 95% had a mean Deyo–Charlson Comorbidity Index of over 3. In this study, all the participants were adult declarants who participated in ACP on their own volition. Other studies have revealed the factors influencing the declarants’ signing of ADs, which include their age, race, whether the declarant was suffering from a disease, socioeconomic status, education level, level of disability, and whether the declarant has knowledge about ADs and end-of-life care [27,28,29,30,31,32].

When Taiwanese ponder death-related issues, they are concerned about themselves and their families. On the personal level, most people wish to die with dignity, and are concerned about their quality of life in lieu of their lifespan. They also expect to understand in advance the medical treatment and care options available for them when they reach the end-of-life stage. On the family level, they do not wish to hand over the major responsibility of making medical decisions to their families, nor do they want to become a social and economic burden to their families. In Taiwan, even though the Patient Right to Autonomy Act has been promulgated, its implementation is not yet thorough [12]. Most patients at present have yet to undergo ACP discussions and AD signing. As of September 2020, only 0.069% (16,360/23,574,334) of Taiwanese aged above 20 years have signed an AD [18,33]. An investigation into the hardships and barriers of implementing ACP showed that most people were willing to participate in ACP due to having experiences in undergoing medical treatments, being informed that they do not have much time to live, wishing their families to understand their opinions, or being hospitalized for severe illness. Nonetheless, some people were highly uncertain about the course of their disease, and perceived that after participating in ACP and signing an AD they would lose the opportunity to try other medical and care options that may give them a chance to live. Some people had an emotional fear of discussing death and refused to participate in ACP. In addition, some people perceived that in the acute phase of their disease, their physical state is highly deteriorated, such that it is inappropriate to discuss ACP, while some perceived otherwise [32,34]. Another study explored the factors that affect whether physicians would undergo ACP with their patients. The results show that physicians prioritized factors such as the patients’ values, their preferences for treatment, and the possibility of unnecessary or unexpected hospitalizations [19]. In terms of systematic factors, the declarants’ education level, structured guidance, and continuous follow-ups were found to play a role too [35].

Even though the differences were not statistically significant, a slightly higher number of female declarants had a designated HCA than males. Compared with those over 60 years of age, the proportion of declarants under 60 years of age with a designated HCA was clearly higher. Based on previous studies, 29.8% of patients over 60 years old needed an HCA towards the end of their life. However, it is difficult to foresee which groups will require an HCA [5]. The study, however, only focused on patients over 60 years old, and the need for an HCA among patients below 60 years old remains unknown. The main reasons for needing an HCA is to express a patient’s wishes when they lose their medical decision-making capacity towards the end of their life, which happens in up to 95% of critically ill patients [17]. The primary factors that lead to such circumstances include cognitive impairment, cerebrovascular disease, and residing in a nursing home [5,36]. We postulate that that a higher proportion of younger patients had a designated HCA because they expected to live longer, were uncertain about the course of future treatment, and lacked experiences that support them in making decisions immediately. In addition, female declarants, those under 60 years of age, and those with a designated HCA required more time during their ACP compared to other groups. According to past studies, the mean length of conversations on ADs was 5.6 min and ranged from 0.9 to 15.0 min, during which most of the time (3.9 min) was spent by the physician in explaining the reasons for initiating AD-related discussions [37]. Medical ethics and physician–patient communication experts could require even more time (mean = 14.7 min; range = 7.4 to 26.3 min) in order to establish relationships, allow the patient to speak more, and discuss social and psychological issues [38]. Yet, to the best of our knowledge, very few studies have analyzed the declarants’ reasons for designating an HCA and spending more time in their ACP process from the perspectives of the declarants themselves, and hence, further research should be invested in this area.

Economic subsidies may help increase the public’s willingness to seek ACP discussions [39]. This trend is evident in our study. In order to promote ACP, our hospital fully reimbursed the cost of ACP for veterans, low-income groups, and hospital employees. As a result, almost half of the declarants in 2019 completed their ACP free of charge. For those who paid for their ACP process, a higher number of declarants had participated in ACP sessions when the cost was lower (NT$1000). Other studies delineated the measures for promoting ACP, such as enhancing the physicians’ skills, increasing their ability to foresee the course of the patients’ disease, strengthening long-term physician–patient relationships, providing good settings for holding discussions, and providing economic subsidies for physicians who have underwent ACP discussions [20,40].

Our study suffers from some limitations. First, there is a lack of complete data pertaining to marital status. Many declarants had not disclosed their marital status when they filled out their basic data, which led to the high number of unspecified marital statuses. Second, there was no further qualitative analysis for the subgroups in this study, and hence, the reasons for the characteristics specific to a subgroup cannot be deduced. In the future, in-depth qualitative research can be performed in this field in order to illuminate more information on certain subgroups.

## 5. Conclusions

Even though the declarants’ gender, age, marital status, and designation of an HCA had affected their motivations for participating in ACP, most of the Taiwanese declarants had sought ACP discussions in the hopes that they could die with dignity. They were concerned about their quality of life, and hoped that they could learn in advance about the end-of-life treatment and care models that fit their wishes. Next, most of them were reluctant to let their family members take on the responsibility of making decisions and did not want to affect their family members on social and economic levels. At present, the proportion of Taiwanese who have participated in ACP discussions and signed their ADs is still low. The provision of economic subsidies could be a means of enhancing their willingness to seek ACP discussions of their own accord. More research should be done to understand the common barriers among subgroups to participating in ACP, as well as their reasons for designating an HCA.

## Figures and Tables

**Table 1:** The demographic characteristics of advance care planning participants in Taipei Veterans General Hospital in 2019.

	Number	Percentage (*n* = 946)
Gender		
Male	406	42.9%
Female	540	57.1%
Age		
20–29 years	17	1.8%
30–39 years	45	4.8%
40–49 years	101	10.7%
50–59 years	152	16.1%
60–69 years	235	24.8%
70–79 years	194	20.5%
80 years and above	202	21.4%
Marital status		
Single	121	12.8%
Married	398	42.1%
Other *	427	45.1%
How many persons participate in each ACP session		
One person	100	10.6%
Two persons	226	23.9%
Three persons and above	620	65.5%
Accompanying relatives		
Came alone	99	10.5%
First or second degree relatives	702	74.2%
Relatives other than first or second degree	8	0.8%
Friends	137	14.5%
Cost		
Free+	468	49.5%
NT$3000	23	2.4%
NT$1500	104	11.0%
NT$1000	351	37.1%
Whether an AD was completed on the spot		
Completed on the spot	596	63.0%
Not completed on the spot	341	36.0%
Not written ^#^	9	1.0%
Time of AD completion		
Completed within 90 days	854	90.2%
Completed after 90 days	37	3.9%
Not completed ^#^	55	5.8%
Designation of an HCA		
Yes	35	3.7%
No	911	96.3%

* Other includes widowed, divorced, and not specified. + The ACP is free of charge for declarants who identify as a veteran, low-income group, or VGHTPE employees who participated in groups of three and above. # The ‘Not written’ and “Not completed’ subgroups include two people who were found to have cognitive impairment during the ACP process.

**Table 2:** The motivations of advance care planning participants in Taipei Veterans General Hospital in 2019 *.

	Suffering from a Disease	Having a Family Member Who Is Suffering from a Disease	Being Single	Reluctant to Let Family Members Take on the Responsibility of Making Decisions	Looking Forward to Dying with Dignity	Making End-Of-Life Pre-Arrangements	Fear of Being a Social and Economic Burden to Family Members	Media Reports and Dissemination	Other	*p*-Value ^$^
Gender										
Male (%, *n* = 1206)	40 (3.3)	31 (2.6)	79 (6.6)	211 (17.5)	302 (25)	269 (22.3)	215 (17.8)	55 (4.6)	4 (0.3)	<0.001
Female (%, *n* = 1712)	57 (3.3)	69 (4.0)	115 (0.9)	345 (20.2)	427 (24.9)	414 (24.2)	327 (19.1)	51 (3.0)	7 (0.4)	
Age										
20-59 years (%, *n* = 1041)	25 (2.4)	37 (3.6)	73 (7.0)	200 (19.2)	239 (23.0)	250 (24.6%)	175 (16.8)	33 (3.2)	9 (0.9)	0.012
≥60 years (%, *n* = 1977)	72 (3.6)	63 (3.2)	121 (6.1)	356 (18.0)	490 (24.8)	433 (21.9)	367 (18.6)	73 (3.7)	2 (0.1)	
Marital Status										
Single (%, *n* = 429)	13 (3.0)	17 (3.0)	87 (20.3)	64 (14.9)	87 (20.3)	90 (21.0)	58 (13.5)	13 (3.0)	0 (0)	<0.001
Married (%, *n* = 1358)	46 (3.4)	41 (3.0)	53 (3.9)	276 (20.3)	319 (23.5)	280 (20.6)	283 (20.8)	59 (4.3)	1 (0.1)	
Other ^+^ (%, *n* = 1231)	38 (3.1)	42 (3.4)	54 (4.4)	216 (17.5)	323 (26.2)	313 (25.4)	201 (16.3)	34 (2.8)	10 (0.8)	
Designation of an HCA										
Yes (%, *n* = 105)	6 (5.7)	2 (1.9)	6 (5.7)	16 (15.2)	26 (24.8)	28 (26.7)	15 (14.3)	3 (2.9)	3 (2.9)	0.003
No (%, *n* = 2893)	91 (3.1)	98 (3.4)	188 (6.5)	540 (18.7)	703 (24.3)	655 (22.6)	527 (18.2)	83 (2.9)	8 (0.3)	

* The number in this table represented the times an option was ticked. The ‘n’ varied in each subgroup because a declarant could tick multiple options about motivations. For example, the option ‘suffering from a disease’ was selected 40 times in all male declarants. ‘*n* = 1206′ in the male subgroup meant male declarants ticked at least one of the nine motivation options 1206 times in sum. ^$^ The *p* values here were calculated by chi-squared tests. ^+^ Other includes widowed, divorced, and not specified.

**Table 3:** The demographic characteristics of advance care planning participants with health care agents in Taipei Veterans General Hospital in 2019.

	Yes	No	*p*-Value
Gender			
Male (%, *n* = 406)	11 (2.7)	395 (97.3)	0.162
Female (%, *n* = 540)	24 (4.4)	516 (95.6)	
Age			
20–59 years (%, *n* = 315)	19 (6.0)	296 (94.0)	0.007
60 years and above (%, *n* = 631)	16 (2.5)	615 (97.5)	
Marital status			
Single (%, *n* = 121)	9 (7.4)	112 (92.6)	0.042
Married (%, *n* = 398)	10 (2.5)	388 (97.5)	
Other * (%, *n* = 427)	16 (3.7)	411 (96.3)	

* Other includes widowed, divorced, and not specified.

**Table 4:** The time required in advance care planning in Taipei Veterans General Hospital in 2019.

	<90 min	>90 min	*p*-Value
Gender			
Male (%, *n* = 406)	393 (96.8)	13 (3.2)	0.004
Female (%, *n* = 540)	499 (92.4)	41 (7.6)	
Age			
20–59 years (%, *n* = 315)	290 (92.1)	25 (7.9)	0.037
60 years and above (%, *n* = 631)	602 (95.4)	29 (4.6)	
Designation of an HCA			
Yes (%, *n* = 35)	28 (80.0)	7 (20.0)	0.001
No (%, *n* = 908)	861 (94.8)	47 (5.2)	
Cost			
Free (%, *n* = 468)	458 (97.9)	10 (2.1)	<0.001
NT$3000 (%, *n* = 23)	23 (100.0)	0 (0)	
NT$1500 (%, *n* = 104)	100 (96.2)	4 (3.8)	
NT$1000 (%, *n* = 351)	311 (88.6)	40 (11.4)	

**Table 5:** The cost of advance care planning participants in Taipei Veterans General Hospital in 2019.

	Free	NT$3000	NT$1500	NT$1000	*p*-Value
Gender					
Male (%, *n* = 406)	259 (63.8)	4 (1.0)	34 (8.4)	109 (26.8)	<0.001
Female (%, *n* = 540)	209 (38.7)	19 (3.5)	70 (13.0)	242 (44.8)	
Age					
20–59 years (%, *n* = 315)	89 (28.2)	8 (2.5)	38 (12.1)	180 (57.1)	<0.001
60 years and above (%, *n* = 631)	379 (60.1)	15 (2.4)	66 (10.5)	171 (27.1)	
Marital status					
Single (%, *n* = 121)	43 (35.5)	9 (7.4)	15 (12.4)	54 (44.6)	<0.001
Married (%, *n* = 398)	198 (49.7)	4 (1.0)	54 (13.6)	142 (35.7)	
Other * (%, *n* = 427)	227 (53.2)	10 (2.3)	35 (8.2)	155 (36.3)	

* Other includes widowed, divorced, and not specified.

## Data Availability

Data is contained within the article.
